# Surface antigen serocleared hepatitis B virus infection increases the risk of mixed cryoglobulinemia vasculitis in male patients with chronic hepatitis C

**DOI:** 10.3389/fimmu.2024.1411146

**Published:** 2024-07-11

**Authors:** Anna Morrone, Valerio Fiorilli, Lilia Cinti, Piergiorgio Roberto, Alejandro L. Ferri, Marcella Visentini, Alessandro Pulsoni, Francesca Romana Spinelli, Adriano De Santis, Guido Antonelli, Stefania Basili, Maria Elena Tosti, Fabrizio Conti, Milvia Casato

**Affiliations:** ^1^ Division of Gastroenterology and Hepatology, Department of Translational and Precision Medicine, Sapienza University of Rome, Rome, Italy; ^2^ Division of Rheumatology, Department of Internal Clinical Sciences, Anaesthesiologic and Cardiovascular Sciences, Sapienza University of Rome, Rome, Italy; ^3^ Laboratory of Microbiology and Virology, Department of Molecular Medicine, Sapienza University of Rome, Rome, Italy; ^4^ The PhD National Programme in “Innovazione Nella Diagnosi, Prevenzione e Terapia Delle Infezioni a Rischio Epidemico-Pandemico”, Department of Medical Biotechnologies, University of Siena, Siena, Italy; ^5^ The PhD National Programme in One Health Approaches to Infectious Diseases and Life Science Research, Department of Public Health, Experimental and Forensic Medicine, University of Pavia, Pavia, Italy; ^6^ Division of Clinical Immunology, Department of Translational and Precision Medicine, Sapienza University of Rome, Rome, Italy; ^7^ Department of Hematology, Sapienza University of Rome, Santa Maria Goretti Hospital, Latina, Italy; ^8^ Department of Molecular Medicine, Laboratory of Microbiology and Virology, Policlinico Umberto I Hospital, Sapienza University of Rome, Rome, Italy; ^9^ Istituto Superiore di Sanità, National Centre for Global Health, Rome, Italy

**Keywords:** HBsAg seroclearance, occult HBV infection, chronic hepatitis C, mixed cryoglobulinemia vasculitis, risk factor

## Abstract

Mixed cryoglobulinemia vasculitis (MCV) is caused in ~90% of cases by chronic hepatitis C virus (HCV^pos^MCV) and more rarely by hepatitis B virus (HBV) infection, or apparently noninfectious. HCV^pos^MCV develops in only ~5% of patients with chronic hepatitis C (CHC), but risk factors other than female gender have not been identified so far. We conducted a retrospective case control study investigating whether past active HBV infection, defined by hepatitis B surface antigen (HBsAg) seroclearance and anti-core antibody (HBcAb) positivity, could be a risk factor for developing HCV^pos^MCV. The prevalence of HBsAg seroclearance was 48% within 123 HCV^pos^MCV patients and 29% within 257 CHC patients (p=0.0003). Multiple logistic regression including as variables gender, birth year, age at HBV testing, cirrhosis, and hepatocellular carcinoma, confirmed an association of HBsAg seroclearance with HCV^pos^MCV [adjusted odds ratio (OR) 2.82, 95% confidence interval (95% CI) 1.73-4.59, p<0.0001]. Stratification by gender, however, showed that HBsAg seroclearance was associated with HCV^pos^MCV in male [OR 4.63, 95% CI 2.27-9.48, p<0.0001] and not in female patients [OR 1.85, 95% 95% CI 0.94-3.66, p=0.076]. HBsAg seroclearance, and more likely occult HBV infection, is an independent risk factor for HCV^pos^MCV in male CHC patients.

## 1 Introduction

Mixed cryoglobulinemia vasculitis (MCV) is a lymphoproliferative disorder of marginal zone B-cells, producing polyreactive IgM endowed with rheumatoid factor activity, which are nonmalignant but are prone to neoplastic transformation; the pathogenic IgM forms cryoprecipitable immune complexes with endogenous IgG that *in vivo* cause a small vessel vasculitis (1). About 90% of MCV cases are associated with chronic hepatitis C virus (HCV) infection (HCV^pos^MCV), and 5% with surface antigen (HBsAg)-positive chronic hepatitis B virus (HBV) infection; in a minority of cases MCV is apparently not associated with infections (noninfectious MCV) (1, 2).

HCV and HBV can infect B-cells and both can cause, besides MCV, non-Hodgkin lymphoma (NHL) (3). HCV is widely believed to cause MCV and indolent NHL by continual antigenic stimulation, since both disorders can regress after the eradication of infection (1, 4); however, mutations of lymphoma-related genes concur to the pathogenesis of HCV^pos^MCV (5).

Besides MCV and B-cell NHL, chronic HCV infection causes a spectrum of extrahepatic manifestations that include diabetes and cardiovascular disease, which is an important cause of mortality in infected people. Although HCV-associated B-cell lymphoproliferative disorders and cardiovascular disease are driven by different mechanisms, inflammation *vs* antigenic pressure and gene mutation, they share the characteristic of regressing after antiviral therapy as especially illustrated by the recent use of highly effective direct-acting antivirals (4, 6–8). While in the case of HCV-associated cardiovascular disease no risk factor have been identified except for those canonical such as diabetes, smoking and hypertension (9), the fact that only a minority of CHC patients develop HCV^pos^MCV suggests specific risk factor(s) that, however, remain elusive.

It is estimated that, although serum cryoglobulins can be found in as high as 40% of subjects with chronic hepatitis C (CHC), only ~5% of them develop HCV^pos^MCV, but the only risk factor for developing MCV identified so far is female gender (1). Concerning host factors, a genome-wide association study identified a segment on chromosome 6 near the NOTCH4 and MHC class II genes conferring 2.15 times the odds of having HCV^pos^MCV within patients with CHC, but it was impossible to determine which one of the two genes determined this association (10). Concerning HCV-related factors, the claim that variations in a sequence of the hypervariable region 1 of the E2 protein were more prevalent in HCV^pos^MCV than in CHC patients (11) was not confirmed by a later study (12). To our knowledge, no other risk factors for developing HCV^pos^MCV have been suggested.

Some studies have shown that hepatitis B surface antigen (HBsAg) seroclearance (13) and occult HBV infection (OBI) (14–16) are associated with an increased risk of developing B-cell lymphoproliferative disorders, and particularly diffuse large B-cell lymphoma (DLBCL). HBsAg seroclearance is commonly defined by negative HBsAg (HBsAg^neg^) and positive anti-core antibody (HBcAb^pos^) serology, and OBI by the presence of HBV DNA in serum and/or of replication-competent covalently closed circular HBV DNA (cccDNA) in tissues of HBsAg^neg^ subjects (17). Currently available commercial assays detect plasma HBV DNA in ~1% of HBsAg^neg^HBcAb^pos^ subjects intermittently and usually at low levels (17), whereas recent highly sensitive assays detect HBV DNA in up to 40% of these cases (18, 19); furthermore, HBV cccDNA was found in liver tissue in 62% of HBsAg^neg^HBcAb^pos^ subjects (20). Therefore, the HBsAg^neg^HBcAb^pos^ serology is commonly used as surrogate marker of OBI in clinical and epidemiological studies (17).

In this retrospective case control study, we investigated the prevalence of HBsAg seroclearance, defined by HBsAg^neg^HBcAb^pos^ serology, in patients with HCV^pos^MCV compared to patients with CHC. In addition, we explored the prevalence of HBsAg seroclearance in a small group of patients with noninfectious MCV compared to patients with B-cell NHL, rheumatoid arthritis, or miscellaneous diseases. Our results reveal an association of HBsAg seroclearance with the risk of developing HCV^pos^MCV in male but not in female CHC patients and confirm female gender as an independent risk factor.

## 2 Methods

### 2.1 Study populations

This retrospective case control study was conducted at the Sapienza University of Rome Hospital, Rome, Italy. We compared the prevalence of HBsAg seroclearance (HBsAg^neg^HBcAb^pos^ serology) in patients with HCV^pos^MCV *vs* patients with CHC without symptoms or signs of vasculitis.

Two independent clinical research centers collected, blinded to each other, data concerning patients with HCV^pos^MCV and CHC, respectively. Demographic, clinical and virological data of consecutive patients were obtained from internal medical records. The minimum requirements for inclusion in the study were the availability of full clinical information, negativity of HBsAg and of HIV serology, and availability of HBcAb serology. Serological and molecular assays for HBV, HCV and HIV were mostly done at the Central Laboratory of Virology of the Sapienza University Hospital, and rarely at other high-quality laboratories. The tertiary Referral Center for Mixed Cryoglobulinemia enrolled patients with HCV^pos^MCV seen between 1997 and 2022; the Division of Gastroenterology and Hepatology enrolled patients with CHC seen between 1999 and 2022.

The diagnosis of CHC was based on the presence of anti-HCV antibodies and detectable viremia for at least 6 months after the first detection of infection in the absence of any clinical symptom or sign suggesting MCV at the time of diagnosis and throughout the follow-up. The diagnosis of HCV^pos^MCV was based on the presence in CHC patients of serum cryoglobulins and of at least one of the following clinical items: purpura, peripheral neuropathy assessed by electroneurography, chronic skin ulcers, biopsy-proven cryoglobulinemic nephropathy.

The diagnosis of cirrhosis was established through blood test and ultrasound findings: reduced liver protein synthesis (prothrombin time, albumin, cholinesterase), liver stiffness evaluation by elastometry, ultrasonographic signs of portal hypertension (splenomegaly, ascites, portal vein collaterals), esophageal varices at endoscopy examination (21). In some cases, the diagnosis was made by liver biopsy. The diagnosis of hepatocellular carcinoma (HCC) was based on radiological hallmarks (contrast uptake in the arterial phase and washout in the venous/late phase) or liver biopsy according to the EASL guidelines (22). Cirrhosis was present in all patients at the first visit or previously, and none developed it during the follow-up.

A secondary analysis was conducted comparing noninfectious MCV (cases) versus other conditions: miscellaneous diseases, indolent B cell NHLs, and rheumatoid arthritis (controls) (see **Supplementary Material**).

This study was conducted according to the ethical guidelines of the 1975 Declaration of Helsinki. All the laboratory studies were done as part of the routine workup of patients and therefore did not require specific ethical approval. Patient consent was waived since this was a retrospective and de-identified study.

### 2.2 Statistical analysis

The values are expressed as number and percent for categorical variables and as median and range for continuous variables. Birth year and age at HBV testing were adopted as time-related variables since other commonly used variables, such as age at infection or at disease onset, could not be clearly determined especially in CHC patients; birth year is of interest as it could cope with variations over time of HBV endemicity in the study area.

Univariate comparisons were done by the Fisher’s exact test for categorical variables and the Mann-Whitney U test for continuous variables. Multivariate analysis was done by unconditional multiple logistic regression, and adjusted odds ratio (OR), 95% confidence interval (95% CI), and p-value are reported. The independent variables used for between-group comparisons were gender, birth year, age at latest HBV testing, HBcAb serology and, in the case of HCV^pos^MCV *vs* CHC, the presence of cirrhosis and of hepatocellular carcinoma (HCC). Preliminary analysis of data revealed a significant inverse correlation between birth year and age at HBV serological testing, seemingly because elderly patients had undergone HBV tests later in life. Since collinearity between independent variables can distort the model development process in multivariate analysis, a stepwise selection procedure (bidirectional inclusion/elimination) was chosen to select variables to be included in the models. All statistical analyses were performed using the GraphPad Prism version 9.1.2 software or STATA Statistics/Data analysis version 16.1. A p-value of less than 0.05 was considered statistically significant.

## 3 Results

During the study period, 1997-2022, 123 cases with HCV^pos^MCV and 257 with CHC (control group) were enrolled. HCV^pos^MCV cases presented the following clinical items: purpura (82%), peripheral neuropathy assessed by electroneurography (63%), chronic skin ulcers (14%), biopsy-proven cryoglobulinemic nephropathy (9%). Cryoglobulins were classified as type 2 in 73% of cases; 10 patients (8%) had associated a B-cell NHL

**Table 1** shows the univariate statistical comparison of demographic, clinical and virologic characteristics in total and gender stratified groups. Total HCV^pos^MCV cases and CHC controls differed for all the variables tested except for HCV genotype distribution (not shown). HCV^pos^MCV patients were mostly female and older than CHC patients; they had a lower percentage of cirrhosis and HCC, and 48% of them were HBcAb positive compared to 29% within CHC patients.

**Table 1:** Univariate analysis of HCV^pos^MCV and CHC patients, total and stratified by gender. Continuous variables are expressed as median (range).

Group	N. of pts	Female n. (%)	Birth year	Age (y) HBV test	Cirrhosis n. (%)	HCC n. (%)	HBcAb^pos^ n. (%)
Total patients							
HCV^pos^MCV	123	74 (60)	1946 (1928-1984)	64 (25-86)	24 (19.5)	3 (2.4)	59 (48)
CHC	257	118 (46)	1956 (1925-1995)	58 (15-89)	94 (37)	28 (11)	74 (29)
P-value		0.0085	<0.0001	<0.0001	0.0009	0.0043	0.0003
Male patients							
HCV^pos^MCV	49	N/A	1956 (1933-1979)	60 (25-86)	12 (25)	2 (4)	32 (65)
CHC	139	N/A	1958 (1925-1995)	54 (15-89)	59 (42)	17 (12)	44 (32)
P-value			0.208	0.097	0.027	0.166	<0.0001
Female patients							
HCV^pos^MCV	74	N/A	1942 (1928-1984)	70 (34-85)	12 (16)	1 (1)	27 (36)
CHC	118	N/A	1950 (1930-1984)	62 (29-87)	35 (30)	11(9)	30 (25)
P-value			<0.0001	0.0004	0.039	0.031	0.108

N/A, Not Applicable.

Gender stratification (**Table 1**) revealed that HBsAg^neg^HBcAb^pos^ serology was significantly (p<0.0001) more prevalent in males, but not in females, with HCV^pos^MCV compared to gender-matched CHC controls. By contrast, significantly earlier birth year and older age at HBV testing compared to gender-matched CHC controls were observed in females and not in males with HCV^pos^MCV. Furthermore, HCV^pos^MCV females were born significantly earlier than males (**Table 1**), and the age at onset of symptoms/signs of MCV was significantly higher in females than in males [median 62 (range 32-85) *vs* 57 (range 30-83) years, p=0.038].

Within the HCV^pos^MCV cohort, HBcAb^pos^ serology was more prevalent in males than in females (p=0.003) while, remarkably, this gender difference was not observed within the CHC control cohort (p=0.33).

Protective levels of anti-HBs antibodies (HBsAb^pos^) were present in a similar proportion of HBcAb^pos^ patients with HCV^pos^MCV (28/59, 47%) or with CHC (32/74, 43%). HBcAb^neg^HBsAb^pos^ serology was found in 25 patients (5/123 HCV^pos^MCV and 20/257 CHC), all of whom were vaccinated. The prevalence of anti-HBs positivity was similar in HBcAb^pos^ males and females with HCV^pos^MCV (16/32 *vs* 17/27); however, the prevalence of anti-HBs positivity was lower within HBcAb^pos^ males with HCV^pos^MCV than in HBcAb^pos^ males with CHC (16/32 *vs* 34/44, p=0.016), while this was not the case for HBcAb^pos^ females (17/27 HCV^pos^MCV *vs* 18/30 CHC).

HBV DNA was searched only in a small fraction of HBcAb^pos^ subjects and was positive in none of 16 HCV^pos^MCV and in 2/14 CHC patients tested.

Stepwise logistic regression analysis in total and gender-stratified populations is reported in **Table 2**. Within total populations, the ORs were 2.82 (95% CI 1.73-4.59) for HBcAb positivity and 0.52 (95% CI 0.29-0.93) for absence of cirrhosis. The association of HBcAb positivity with HCV^pos^MCV was retained after excluding patients with cirrhosis by univariate (42/99 vs 42/165, p=0.006) and multivariate (OR 2.24, 95% CI 1.29-3.90, p=0.004) analysis (not shown). After gender stratification, HBcAb positivity was associated with HCV^pos^MCV in males (OR 4.63, 95%CI 2.27-9.48, p<0.0001) but not in females.

**Table 2:** Stepwise logistic regression analysis of HCV^pos^MCV and CHC (control group) patients, total and stratified by gender.

Variable	Odds ratio	95% CI	P-value
Total patients			
Gender [f]	1.57	0.97-2.54	0.069
Age HBV test	1.03	1.01-1.05	0.0005
Cirrhosis [yes]	0.52	0.29-0.93	0.027
HCC [yes]	0.29	0.08-1.08	0.066
HBcAb [pos]	2.82	1.73-4.59	<0.0001
Male patients			
Cirrhosis [yes]	0.36	0.16-0.78	0.009
HBcAb [pos]	4.63	2.27-9.48	<0.0001
Female patients			
Birth year	0.95	0.93-0.98	<0.0001
Cirrhosis [yes]	0.58	0.25-1.32	0.194
HCC [yes]	0.15	0.02-1.35	0.091
HBcAb [pos]	1.85	0.94-3.66	0.076

As a corollary to the study in HCV^pos^MCV patients, we performed an exploratory study in a small group (n=39) of patients with noninfectious MCV, a much rarer disorder. Comparator groups were patients with miscellaneous diseases (n=376), indolent B-cell NHL (n=275), or rheumatoid arthritis (n=195); information on the study populations and on statistical analyses is provided in **Supplementary Material**. By stepwise logistic regression (**Supplementary Table 1**), the adjusted ORs for HBcAb positivity were 3.97 (95% CI 1.69-9.32, p=0.002) *vs* miscellaneous diseases, 3.63 (95% CI 1.66-7.91, p=0.001) *vs* indolent NHL, and 2.39 (95% CI 1.05-5.48, p=0.039) *vs* rheumatoid arthritis. The paucity of noninfectious MCV patients did not allow gender stratification.

## 4 Discussion

Patients with HCV^pos^MCV or CHC differed for several of the variables tested. Females were more prevalent among HCV^pos^MCV than CHC patients as known from several studies (23–25), and the prevalence of cirrhosis was lower within HCV^pos^MCV patients as also reported in a large multicenter study from Italy comparing HCV^pos^MCV and CHC patients with asymptomatic serum cryogobulins (25). A lower prevalence of cirrhosis in HCV^pos^MCV than in CHC patients might be due to an earlier onset of vasculitis manifestations than of liver function decompensation, and to the fact that in our study the hepatology center collecting CHC patients is particularly devoted to the care of advanced liver disease.

Birth year and age at HBV testing are important variables since they impact on possible changes over time of HBV prevalence in the study area, on the high rate of false HBcAb positivity with earlier assays (17), and on the length of time of exposure to the risk of infection. The fact that male patients with HCV^pos^MCV and CHC did not differ significantly for these variables reassures about confounding.

Gender preference for adverse outcomes associated with HBsAg seroclearance is not unprecedented. A retrospective cohort study (26) showed that male gender was an independent risk factor of developing HCC after HBsAg seroclearance whereas older age at seroclearance was a risk factor in females. The latter finding may recall our observation that, within the HCV^pos^MCV cohort, females were born earlier and were older at the onset of vasculitis symptoms than males.

A possible explanation for the gender-related risk of HCC and HCV^pos^MCV after HBsAg seroclearance might be a higher prevalence of OBI in males. Indeed, it has been argued (27) that in areas where the male/female ratio of HBV infection in blood donors is about 1 the prevalence of OBI in males ranges from 62% to 88%. A higher prevalence of OBI in males has been reported both in high endemic areas such as South Africa (28) and in medium-low endemic European countries (29).

It is reasonable to assume that the prevalence of HBcAb positivity may roughly parallel that of OBI (17), and that the increased risk of HCV^pos^MCV in HBsAg^neg^/HBcAb^pos^ CHC patients may depend, like in the case of HCC (30), on OBI rather than on HBsAg seroclearance itself. At the state of the art, it is difficult to determine the real prevalence of OBI, since serum HBV DNA is detected by commercial assays in ~1% of HBsAg^neg^HBcAb^pos^ persons while highly sensitive assays and search of cccDNA in liver reveal a much higher prevalence (18–20). We noticed a lower frequency of anti-HBs positivity in HBcAb^pos^ male patients with HCV^pos^MCV rather than with CHC. It has been reported that the probability of having detectable serum HBV DNA is higher in HBsAg^neg^/HBcAb^pos^ individuals who are anti-HBs negative (31, 32) and, interestingly, male sex and absence of anti-HBs are independent risk factors for reactivation of HBV infection in HBsAg^neg^HBcAb^pos^ lymphoma patients treated with rituximab (33). Thus, these observations may suggest that absence of anti-HBs antibodies and male sex concur to increase the risk of OBI, and therefore of MCV, in HBsAg serocleared CHC patients.

Chronic infections by HBV or HCV cause HCC and B-cell NHL by mechanisms that include mutations of tumor-driver genes (5, 34–36). OBI is believed to cause oncogenic mutations in hepatocytes by mechanisms involving integration of viral DNA into the host genome and the production of potentially mutagenic proteins by cccDNA (30); the presence of putatively pro-oncogenic quasispecies of HBV cccDNA in tumor cells suggests a similar mechanism in OBI-associated DLBCL (16).

Recent knowledge on gene mutations in monoclonal B-cells of HCV^pos^MCV patients, although limited, may provide clues to explain a pathogenetic role of OBI in this disease. Although considered non-neoplastic, clonal B-cells of HCV^pos^MCV patients harbor mutations of several lymphoma-related genes (5). These genes regulate NFκB and NOTCH signaling and cell proliferation (*KLF2, TNFAIP3, NOTCH1)*, chromatin organization (*HIST1H2BE, EP300*), cell communication (*FAT1, PTPRD*), and V(D)J mutation (*KLHL6*). Remarkably, some of these genes (*KLF2*, *TNFAIP3, NOTCH1, HIST1H2BE*) are recurrently mutated in HBsAg^pos^ DLBCL (36) and in HCV-associated B-cell NHL (5, 35). OBI increases the risk of HCC in CHC patients, indicating a synergistic effect that may include additive mutagenesis (30); thus, it can be speculated that HBV cccDNA in rogue B-cells of subjects with CHC may cooperate with HCV in inducing gene mutations that drive dysregulated clonal expansion and eventually HCV^pos^MCV. These mutations are seemingly non-transforming, since in most patients HCV^pos^MCV regresses after the clearance of HCV by therapy with interferon (1) or with direct-acting antivirals (8, 37).

Our preliminary data suggest that HBsAg seroclearance can also be associated with the risk of noninfectious MCV. In this regard, it is of interest that also in this disorder the clonally expanded rogue B-cells harbor mutations of lymphoma-related genes (*CARD11, TNFAIP3, CCND3, ID3, BTG2*, *KLHL6*) (38). These genes are also mutated in HBsAg^pos^ DLBCL (36) and in HCV-associated B-cell NHL (5, 35). Remarkably, two genes mutated in noninfectious MCV (*TNFAIP3* and *KLHL6*) are also mutated in HCV^pos^MCV (5). The *KLHL6* gene is particularly interesting, since in noninfectious MCV it has been shown that its mutation dysregulates V(D)J somatic mutation, leading to the generation in rogue B cells of an “iconic” pathogenic antibody carrying the WA public idiotype (38). The WA idiotype is expressed by clonal B-cells of more than half of patients with noninfectious MCV (38) or with HCV^pos^MCV (39), suggesting *KLHL6* mutation as one possible pathogenetic link between these disorders.

To conclude, we first acknowledge that our study suffers from limitations: it was carried in a low HBsAg endemic area (central Italy), asymptomatic cryoglobulins were not investigated in CHC patients, and serum HBV DNA was searched only in a fraction of patients. Nevertheless, our data robustly support an association of HBsAg seroclearance with the risk of developing HCV^pos^MCV in male CHC patients; to the best of our knowledge, this is the first exogenous risk factor for HCV^pos^MCV submitted so far. Studies in different HBV endemic areas and in-depth investigation of the role of OBI are warranted. Also, the possibility that OBI might be a risk factor for “noninfectious” MCV should be pursued by further studies.

## Data availability statement

The original contributions presented in the study are included in the article/**Supplementary Material**. Further inquiries can be directed to the corresponding author.

## Ethics statement

Ethical approval was not required for the study involving humans in accordance with the local legislation and institutional requirements. Written informed consent to participate in this study was not required from the participants or the participants’ legal guardians/next of kin in accordance with the national legislation and the institutional requirements.

## Author contributions

AM: Methodology, Writing – review & editing, Data curation, Investigation. VF: Data curation, Investigation, Methodology, Writing – review & editing. LC: Data curation, Investigation, Writing – review & editing. PR: Data curation, Investigation, Writing – review & editing. AF: Data curation, Investigation, Writing – review & editing. MV: Data curation, Writing – review & editing, Supervision. AP: Data curation, Supervision, Writing – review & editing. FS: Data curation, Supervision, Writing – review & editing. AS: Data curation, Supervision, Writing – review & editing, Methodology. GA: Data curation, Supervision, Writing – review & editing, Methodology. SB: Data curation, Supervision, Writing – review & editing, Funding acquisition. MT: Data curation, Methodology, Supervision, Writing – review & editing, Formal analysis, Software. FC: Data curation, Methodology, Supervision, Writing – review & editing. MC: Methodology, Supervision, Writing – review & editing, Conceptualization, Formal analysis, Writing – original draft.
