# Supplementary material for: Surface antigen serocleared hepatitis B virus infection increases the risk of mixed cryoglobulinemia vasculitis in male patients with chronic hepatitis C
Source: Front Immunol. 2024 Jul 11;15:1411146. doi: 10.3389/fimmu.2024.1411146 (PMC11269149; doi:10.3389/fimmu.2024.1411146)
Supplement: Supplementary file 1 [file DataSheet_1.docx]

***Supplementary Material***

**Study populations: noninfectious MCV and comparator groups**

For each disease group, each of four independent clinical research centers at the Sapienza University Hospital, blinded to each other, collected demographic, clinical and virological data concerning consecutive patients with the disease of competence by reviewing internal medical records and electronic databases. The tertiary Referral Center for Mixed Cryoglobulinemia collected data concerning 39 patients with noninfectious MCV seen between 2001 and 2022. The Central Laboratory of Virology collected from the internal electronic database the data concerning 376 consecutive patients with miscellaneous diseases studied between February and March 2023. The Division of Hematology collected data from 275 consecutive patients with indolent B-cell NHL seen between 1999 and 2022. The Division of Rheumatology collected data from 195 consecutive patients with rheumatoid arthritis seen between 2011 and 2023.

Noninfectious MCV was diagnosed according to established criteria.^1^ The main clinical manifestations of vasculitis were purpura (74%), peripheral neuropathy (59%), chronic skin ulcers (23%), and cryoglobulinemic nephropathy (15%). Cryoglobulins were classified as type 2 in 77% of cases; 3 patients (8%) had associated a B-cell NHL.

The group of miscellaneous diseases (n=376) included patients with cardiovascular disorders (25%), infectious diseases other than HIV, HCV or HBsAg^pos^ HBV infection (24%), surgery (13%), miscellaneous internal medicine diseases (10%), hematologic diseases other than NHL (7%), end-stage renal disease on dialysis (4%), neurological diseases (3%), and other diseases (13%).

The group of indolent B cell NHLs (n=275) included 194 (71%) follicular lymphoma; 42 (15%) marginal zone lymphomas (MZL), of which 39 extranodal (other than splenic) and 3 nodal; 23 (8%) low-grade B cell NHL not otherwise specified; 15 (5%) splenic MZL; 1 lymphoplasmacytic lymphoma.

The group of rheumatoid arthritis included 195 patients; the diagnosis was done according to the criteria of the Italian Group for the Study of Early Arthritis (GISEA).

*References*

1. Terrier B, Krastinova E, Marie I *et al*. Management of noninfectious mixed cryoglobulinemia vasculitis: data from 242 cases included in the CryoVas survey. Blood 2012;119:5996-6004.

**Supplementary Table 1.** Univariate and multivariate (stepwise logistic regression) analysis of noninfectious MCV cases *vs* different comparator groups.

| **Univariate analysis** | | | | | | |
| --- | --- | --- | --- | --- | --- | --- |
| **Group** | N. of  pts | Female  n. (%) | Birth year | Age (y)  HBV test | | HBcAb^pos^ n. (%) |
| **Noninfectious MCV** | 39 | 25 (64) | 1950 (1927-1980) | 63 (24-81) | | 12 (31) |
| **Miscellaneous diseases** | 376 | 157 (42) | 1951 (1938-1962) | 72 (61-85) | | 52 (14) |
| *p-value vs noninf. MCV* |  | *0.0100* | *0.9601* | *0.0001* | | *0.0095* |
|  |  |  |  |  | |  |
| **Indolent NHL** | 275 | 155 (56) | 1951 (1938-1964) | 62 (40-82) | | 30 (11) |
| *p-value vs noninf. MCV* |  | *0.3920* | *0.9767* | *0.4523* | | *0.0019* |
|  |  |  |  |  | |  |
| **Rheumatoid arthritis** | 195 | 157 (81) | 1955 (1930-1996) | 66 (22-87) | | 26 (13) |
| *p-value vs noninf. MCV* |  | *0.0340* | *0.0531* | *0.2855* | | *0.0290* |
| **Multivariate analysis** | | | | | | |
| **Variable** | **Odds ratio** | | **95% CI** | | **P-value** | |
| **Miscellaneous diseases** |  | |  | |  | |
| Gender [f] | 2.76 | | 1.28-5.94 | | 0.0090 | |
| Age at HBV test | 0.84 | | 0.80-0.90 | | <0.0001 | |
| HBcAb [pos] | 3.97 | | 1.69-9.32 | | 0.0020 | |
| **Indolent NHL** |  | |  | |  | |
| HBcAb [pos] | 3.63 | | 1.66-7.91 | | 0.0012 | |
| **Rheumatoid arthritis** |  | |  | |  | |
| Gender [f] | 0.47 | | 0.22-1.01 | | 0.0520 | |
| Birth year | 0.97 | | 0.94-1.00 | | 0.0690 | |
| HBcAb [pos] | 2.39 | | 1.05-5.48 | | 0.0390 | |
